# Checkpoint Pathways in Childhood Cleft Lip and Palate—Associations of PD1 and CTLA4 with Clinical Parameters

**DOI:** 10.3390/ijms27219488

**Published:** 2026-10-24

**Authors:** Margareta Budner, Kamil Torres, Violetta Opoka-Winiarska, Agnieszka Lasota, Małgorzata Gdak, Paulina Mertowska, Sebastian Mertowski, Krzysztof Gosik, Minseung Kang, Tomasz Urbanowicz, Ewelina Grywalska

**Affiliations:** 1 Department of Plastic, Reconstructive and Microsurgery, Medical University of Lublin, ul. Jaczewskiego 8, 20-090 Lublin, Poland; margareta.budner@umlub.edu.pl (M.B.); kamil.torres@umlub.edu.pl (K.T.); 2 Department of Pediatric Pulmonology and Rheumatology, Medical University of Lublin, ul. Prof. Antoniego Gębali 6, 20-093 Lublin, Poland; violetta.opoka-winiarska@umlub.edu.pl; 3 Department of Jaw Orthopaedics, Medical University of Lublin, ul. Chodźki 6, 20-093 Lublin, Poland; agnieszka.lasota@umlub.edu.pl; 4 Department of Pediatric Pulmonology and Rheumatology, University Children’s Hospital, ul. Prof. Antoniego Gębali Street 6, 20-093 Lublin, Poland; 5 Department of Experimental Immunology, Medical University of Lublin, ul. Chodźki 4a, 20-093 Lublin, Polandkrzysztof.gosik@umlub.edu.pl (K.G.); ewelina.grywalska@umlub.edu.pl (E.G.); 6 College of Medicine, Korea University, 73 Goryeodae-ro, Seongbuk-gu, Seoul 02841, Republic of Korea; vickyk03@korea.ac.kr; 7 Cardiac Surgery and Transplantology Department, Poznan University of Medical Sciences, ul. Długa 1/2, 61-848 Poznań, Poland; tomasz.urbanowicz@skpp.edu.pl; 8 Department of Precision Medicine in Medical Surgical and Critical Area, University of Palermo, 90134 Palermo, Italy

**Keywords:** cleft lip, cleft palate, immune checkpoints, programmed cell death 1 receptor, PD-L1, CTLA-4, immunophenotyping, pediatric immunology

## Abstract

Cleft lip and/or palate (CL/P) is associated with recurrent upper respiratory tract complications, yet the immunoregulatory mechanisms accompanying this defect remain insufficiently understood. This study aimed to characterize cellular and soluble parameters associated with PD-1, PD-L1, and CTLA-4 in children with CL/P and to analyze their associations with selected clinical and hematological characteristics. This single-center cross-sectional study included 32 children with CL/P and 16 healthy children as controls. Serum concentrations of sPD-1, sPD-L1, and sCTLA-4 were measured, and the percentages of cells expressing PD-1, PD-L1, and CTLA-4 within T-lymphocyte (CD3+ CD4+ and CD3+ CD8+) and B-lymphocyte (CD19+) populations were assessed using flow cytometry. We analyzed relationships between immunological and clinical parameters, as well as differences between subgroups by gender and mode of delivery. Children with CL/P exhibited significantly higher serum concentrations of sPD-1, sPD-L1, and sCTLA-4, as well as higher percentages of T and B lymphocytes expressing these immune checkpoint molecules, compared to the control group (*p* < 0.0001 in all cases). We observed significant associations among selected immune checkpoint-related parameters, including a positive correlation between sPD-1 and sCTLA-4 and an inverse correlation between the percentages of CD19+ PD-L1+ and CD19+ CTLA-4+ B lymphocytes. The markers clearly distinguished children with CL/P from healthy children in the study cohort; however, we found no consistent differences by gender or mode of delivery. Children with CL/P exhibited distinct cellular and soluble immune checkpoint-related profiles compared to the control group. However, given the limited sample size and the study’s cross-sectional design, these results should be considered preliminary and require confirmation in larger, independent cohorts.

## 1. Introduction

Cleft lip and/or palate (CL/P) is among the most common congenital craniofacial anomalies, affecting approximately 1–2 per 1000 live births worldwide, although its frequency varies considerably by geographic region and population. In Europe, the pooled incidence has been estimated at approximately 1.6 per 1000 live births [1,2,3]. Isolated cleft palate is less common than cleft lip with or without cleft palate, with reported incidence estimates varying considerably across populations, from approximately 1.3 to 25.3 per 10,000 live births [4,5]. Orofacial clefts comprise a heterogeneous group of defects that differ in anatomical extent, clinical presentation, and etiology. Their development is multifactorial and involves interactions between genetic susceptibility and environmental exposures [6,7,8,9]. Environmental factors associated with an increased risk include maternal smoking, alcohol consumption, and inadequate folate or vitamin supplementation during early pregnancy [10,11].

The clinical consequences of CL/P extend beyond the craniofacial defect itself and vary depending on the cleft phenotype. Affected children may experience feeding difficulties, impaired velopharyngeal function, Eustachian tube dysfunction, and otitis media, frequently requiring multidisciplinary care and repeated medical interventions [12,13,14]. Population-based studies have also reported an increased risk of respiratory infections and related hospitalizations among children with CL/P compared with unaffected children [15,16]. In addition, CL/P may be associated with broader long-term clinical and psychosocial consequences extending beyond childhood [17,18,19,20]. Even less apparent phenotypes, such as submucous cleft palate, may be associated with functional abnormalities, including velopharyngeal insufficiency and nasal regurgitation [21]. Collectively, these clinical manifestations may contribute to recurrent local irritation, infectious episodes, and repeated inflammatory stimulation within the upper respiratory tract. However, the extent to which these processes are associated with systemic immune-regulatory alterations remains insufficiently understood. Despite the relatively high burden of respiratory and otologic complications in children with CL/P, their systemic immune-regulatory characteristics remain insufficiently understood. Recurrent infections and repeated antigenic exposure may influence immune activation and regulatory pathways, but the extent to which these processes are reflected in peripheral immune phenotypes remains poorly established. Chronic and recurrent inflammatory stimulation is known to affect immune homeostasis and the balance between activating and inhibitory signaling pathways [16,22,23,24].

Immune checkpoints regulate lymphocyte activation, proliferation, effector functions, and peripheral immune tolerance. Cytotoxic T-lymphocyte-associated protein 4 (CTLA-4) is closely linked to the CD28 costimulatory axis because it competes with CD28 for the B7 family ligands CD80 and CD86, limiting excessive T-cell activation [25,26,27,28,29]. Another major regulatory pathway involves programmed cell death protein 1 (PD-1) and its ligands, programmed death-ligand 1 (PD-L1; CD274) and programmed death-ligand 2 (PD-L2; CD273), which contribute to the regulation of T-cell, B-cell, and NK-cell responses [30,31,32,33]. PD-1 signaling can limit lymphocyte activation, proliferation, and cytokine production and may be modulated under conditions of sustained antigenic exposure and immune activation [34,35].

However, data concerning PD-1/PD-L1- and CTLA-4-related immune regulation in children with CL/P remain limited. Therefore, it is unclear whether cellular and soluble immune-checkpoint parameters differ between children with clefts and healthy children, or whether they associate with clinical, developmental, and hematological characteristics.

The aim of this study was to compare cellular and soluble PD-1, PD-L1, and CTLA-4-related parameters between children with CL/P and healthy controls. We assessed surface expression of these molecules in CD4+ and CD8+ T lymphocytes and CD19+ B lymphocytes, along with serum concentrations of their soluble forms. We additionally evaluated associations between immune-checkpoint parameters and selected clinical and hematological variables, including sex and mode of delivery.

## 2. Results

### 2.1. Baseline Characteristics and Differences in Immunoglobulin Levels, Blood Counts, and Lymphocyte Subsets Between Children with CL/P and Healthy Controls

The study included 32 children with CL/P and 16 healthy controls. Mean age was comparable between groups (9.44 ± 4.23 vs. 9.81 ± 3.56 years, respectively; *p* = 0.7078). Sex distribution was also similar, with boys accounting for 17 of 32 participants (53.1%) in the CL/P group and 9 of 16 (56.3%) in the control group. However, selected perinatal characteristics differed. Cesarean delivery was more frequent among children with CL/P than among controls (59.4% vs. 25.0%; *p* = 0.0339), whereas a history of preterm birth was more common in the control group (43.8% vs. 12.5%; *p* = 0.0270). Birth weight also differed between the groups, with median values of 3380 g in children with CL/P and 3039 g in controls (*p* = 0.0397). These findings indicate that, despite comparable age and sex distributions, the investigated groups were not fully balanced with respect to perinatal characteristics. We compared non-Spanish Caucasian children with CL/P (*n* = 32) with healthy controls (*n* = 16) for immunoglobulin concentrations, peripheral blood parameters, and leukocyte subpopulations. Children with clefts showed significantly lower IgA and IgE concentrations, whereas IgM and IgG did not differ significantly between groups. In peripheral blood, group. Platelet, total leukocyte, and lymphocyte counts did not differ significantly. Flow cytometric analysis demonstrated significantly lower percentages of CD45+ leukocytes, CD3+ T lymphocytes, CD3+ CD4+ T lymphocytes, and CD19+ B lymphocytes in children with CL/P compared with healthy controls. In contrast, the proportion of CD3+ CD8+ T lymphocytes did not differ significantly between groups (Table 1).

### 2.2. Increased Frequencies of Checkpoint-Positive Lymphocytes and Elevated Soluble Checkpoint Levels in Children with CL/P: Comparisons by Sex and Delivery Mode

Children with CL/P (*n* = 32) exhibited significantly higher proportions of PD-1-, PD-L1-, and CTLA-4-positive cells within the CD3+ CD4+ and CD3+ CD8+ T lymphocyte populations and CD19+ B lymphocytes compared with healthy controls (*n* = 16; all *p* < 0.0001). In addition, serum concentrations of the soluble immune checkpoint molecules sPD-1, sPD-L1, and sCTLA-4 were significantly elevated in the CL/P group (all *p* < 0.0001). These findings indicate differences in cellular and soluble immune checkpoint-related parameters between the investigated groups (Table 2).

In the gender-stratified analysis (boys, *n* = 17; girls, *n* = 15), no significant differences were observed in soluble immune-checkpoint concentrations or in the proportions of CD4+, CD8+, and CD19+ lymphocytes expressing PD-1, PD-L1, or CTLA-4. Only non-significant trends toward higher sPD-L1 levels and a higher proportion of CD8+ PD-L1+ T lymphocytes were observed in boys. Table 3 presents detailed data.

In the analysis stratified by delivery mode (vaginal delivery, *n* = 13; cesarean section, *n* = 19), no significant differences were observed in soluble immune-checkpoint concentrations or in most lymphocyte checkpoint phenotypes. The only significant difference concerned CD19+ PD-L1+ B lymphocytes, which were more frequent in children born by cesarean section (*p* = 0.0493). Table 4 presents the detailed results.

### 2.3. Correlations Among Clinical, Hematological, and Immune Checkpoint Parameters in the CL/P Cohort and Its Subgroups

In the cleft cohort, correlation analysis revealed several expected physiological relationships between developmental and hematological parameters. Age, height, and body weight were strongly positively associated, while leukocyte count correlated closely with neutrophil count and erythrocyte count with hemoglobin concentration. In addition, age and body size showed inverse associations with lymphocyte count, consistent with age-related changes in peripheral leukocyte distribution within the pediatric population (Supplementary Table S1).

We also identified several significant relationships among immune-checkpoint parameters. Soluble checkpoint markers showed positive associations with each other, particularly sPD-1 with sCTLA-4. Soluble PD-1 and PD-L1 levels were also positively associated with selected checkpoint-expressing CD8+ T-cell subsets. We also observed correlations between checkpoint phenotypes across T- and B-cell compartments. In contrast, we found a strong inverse association between the proportions of PD-L1+ and CTLA-4+ cells within the CD19+ B-cell population. These findings indicate that checkpoint-related parameters were not independent, although correlation analysis alone cannot determine their biological significance (Supplementary Table S1).

Gender-stratified analyses showed broadly similar patterns in boys and girls. In both groups, age, height, and body weight were strongly correlated, as were leukocyte and neutrophil counts and erythrocyte counts and hemoglobin concentration. Selected immune-checkpoint associations were also present in both sexes, including a positive relationship between CD4+ PD-L1+ and CD4+ CTLA-4+ T-cell fractions. Overall, the correlation structure was largely comparable between boys and girls, with developmental and hematological relationships predominating in both subgroups (Supplementary Tables S2 and S3; Table 5).

Correlation analysis stratified by delivery mode revealed several relationships common to both vaginally delivered and cesarean-born children. In both groups, erythrocyte count and hemoglobin concentration were strongly associated; CD3+ and CD8+ lymphocyte percentages were strongly associated with age, height, and body weight; and leukocyte and neutrophil counts were strongly associated (Table 6).

However, selected correlations differed between delivery-mode subgroups. In particular, the association between birth weight and IgG concentration was opposite: negative in children born vaginally and positive in those born by cesarean section. We observed a positive correlation between sPD-1 and sCTLA-4 in both groups, but it was stronger among children delivered by cesarean section. These subgroup-specific findings should be interpreted cautiously given the limited sample sizes. Additional statistically significant correlations are presented in Supplementary Tables S4 and S5.

### 2.4. Exploratory ROC Analyses of Immune Checkpoint Markers: CL/P Versus Healthy Controls and Subgroup Comparisons

We performed receiver operating characteristic (ROC) analysis to evaluate the ability of the investigated immune checkpoint-related parameters to distinguish children with CL/P from healthy controls in the study cohort. All three soluble markers, sPD-1, sPD-L1, and sCTLA-4, had an observed area under the ROC curve (AUC) of 1.00, while the cellular markers had AUC values ranging from 0.92 to 1.00 (Table 7). These findings indicate pronounced between-group discrimination in the analyzed dataset. However, given the limited sample size and the absence of independent validation, the reported AUC values should be interpreted as exploratory, within-sample estimates rather than indicators of established diagnostic performance.

Within the cleft cohort, ROC analysis showed that the evaluated markers did not significantly discriminate by gender or delivery mode. AUC values were generally low to moderate and not statistically significant, except for CD19+ PD-L1+ cells, which showed modest discrimination by delivery mode (AUC = 0.7085; *p* = 0.0482). Table 8 and Table 9 present detailed results. Given the small subgroup sizes, these findings should be interpreted cautiously.

## 3. Discussion

The present study identified several differences in immunological parameters between children with CL/P and healthy pediatric controls. Children with CL/P exhibited lower proportions of CD3+ T lymphocytes, CD3+ CD4+ T lymphocytes, and CD19+ B lymphocytes, whereas the proportion of CD3+ CD8+ T lymphocytes did not differ significantly between groups. These findings were accompanied by lower serum IgA and IgE concentrations, while IgG and IgM concentrations remained comparable. In contrast, the proportions of PD-1-, PD-L1-, and CTLA-4-positive cells within the investigated T- and B-lymphocyte populations, as well as the serum concentrations of their soluble counterparts, were significantly higher in children with CL/P. Correlation analyses additionally revealed several relationships among immune checkpoint-related parameters and expected associations between developmental and hematological variables, including age, height, body weight, leukocyte and neutrophil counts, and erythrocyte count and hemoglobin concentration. Together, these observations indicate differences in selected immune phenotypes and immune checkpoint-related profiles in the investigated cohort, although their biological and clinical significance remains to be established.

Previous studies investigating systemic immunological characteristics in children with CL/P have reported alterations in lymphocyte subpopulations, although the available evidence remains limited. Timokhina et al. [23] evaluated 74 children with congenital CL/P, including 43 patients with unilateral and 31 with bilateral complete clefts, and examined their immunological profiles in association with oral cavity disorders. The authors observed significantly lower proportions of CD4+ and CD8+ T lymphocytes and higher proportions of CD22+ B lymphocytes in children with bilateral clefts than in those with unilateral defects. Our findings partially align with these observations, particularly regarding differences in CD4+ T lymphocytes. However, unlike the results reported by Timokhina et al., we did not observe a significant difference in the proportion of CD3+ CD8+ T lymphocytes between children with CL/P and healthy controls, while the proportion of CD19+ B lymphocytes was significantly lower in the CL/P group. These differences should be interpreted in the context of distinct study designs, comparison groups, clinical characteristics, and immunophenotyping approaches. In particular, Timokhina et al. compared children with different anatomical manifestations of CL/P, whereas our study compared CL/P with healthy pediatric controls. Moreover, the two studies used different markers for identifying B lymphocytes (CD22 and CD19, respectively). Nevertheless, both investigations suggest that alterations in peripheral lymphocyte populations may accompany CL/P, although their direction and magnitude may vary by population and clinical characteristics.

The observed differences in serum immunoglobulin concentrations offer additional insight into the immune characteristics of children with CL/P. In our study, IgA and IgE concentrations were significantly lower than in healthy controls, whereas IgG and IgM did not differ significantly. These findings may reflect heterogeneity in selected components of humoral immunity; however, they should not be interpreted as evidence of clinically relevant antibody deficiency. Serum immunoglobulin concentrations vary with age and individual immunological characteristics, so interpretation requires age-specific reference ranges. Furthermore, our study did not measure antigen-specific antibody responses or assess B-lymphocyte function. Therefore, the observed differences in IgA and IgE remain descriptive and require confirmation in larger pediatric cohorts.

The potential relevance of these immunological findings should be considered in the broader clinical context of CL/P. Previous epidemiological studies have demonstrated an increased burden of respiratory and otologic complications among affected children. Laager et al. [15], in a large population-based cohort study, reported higher hospitalization rates for respiratory infections among children with CL/P than among unaffected children. Similarly, Kurosaka et al. [16] identified an increased risk of otitis media in selected CL/P phenotypes and reported an association between CL/P and upper respiratory tract inflammation in the overall investigated population. These observations are consistent with previous reports emphasizing the importance of Eustachian tube dysfunction and recurrent middle-ear inflammation in children with palatal clefts [14]. Recurrent exposure to infectious and inflammatory stimuli may represent one of several factors contributing to variability in immune activation and regulatory pathways. Nevertheless, anatomical and functional abnormalities associated with CL/P may independently contribute to recurrent infections, and such complications do not necessarily indicate systemic immune dysfunction. Importantly, our study did not systematically assess infection history, microbiological parameters, or the frequency and severity of respiratory complications. Consequently, we cannot directly attribute the observed differences in immune checkpoint-related parameters to recurrent infections or chronic inflammatory stimulation.

A key finding of our study was the significantly higher proportion of PD-1-, PD-L1-, and CTLA-4-positive cells in the T- and B-lymphocyte populations we investigated. PD-1/PD-L1 and CTLA-4 represent major immunoregulatory pathways involved in controlling lymphocyte activation, proliferation, effector responses, and peripheral immune tolerance [28,30,31,33,35]. PD-1 signaling contributes to the regulation of lymphocyte responses following antigenic stimulation, whereas CTLA-4 limits T-cell activation through its interactions with the CD28 costimulatory pathway. Previous studies show that inhibitory immune checkpoint expression may change during sustained antigenic exposure, chronic infection, and prolonged immune activation [31,35,36,37]. However, interpreting increased checkpoint expression depends on the cellular context and functional state of the lymphocytes studied. In particular, increased PD-1 expression is not necessarily equivalent to lymphocyte exhaustion, as this molecule may also be expressed by activated T lymphocytes. Similarly, increased CTLA-4 expression does not independently demonstrate enhanced suppressive activity. Therefore, the higher frequencies of checkpoint-positive lymphocytes observed in our cohort should be interpreted as phenotypic differences rather than direct evidence of impaired immune function or increased inhibitory signaling.

In addition to cellular immune checkpoint expression, we observed significantly elevated serum concentrations of sPD-1, sPD-L1, and sCTLA-4 in children with CL/P. Correlation analyses revealed positive associations among selected soluble checkpoint-related parameters, particularly between sPD-1 and sCTLA-4. We also identified additional relationships between checkpoint-positive T- and B-lymphocyte populations. Notably, we observed a strong inverse correlation between the proportions of PD-L1-positive and CTLA-4-positive cells within the CD19+ B-lymphocyte population. These findings indicate that selected checkpoint-related parameters were statistically associated within the investigated cohort. However, correlation analyses alone cannot establish the functional significance of these relationships. In particular, correlations between soluble immune checkpoint molecules do not demonstrate coordinated signaling activity or identify their cellular sources. Likewise, the inverse association between PD-L1-positive and CTLA-4-positive B-lymphocyte fractions does not establish direct regulatory interactions between these molecules. Although the biological functions of PD-1/PD-L1 and CTLA-4 have been extensively investigated in other immunological and pathological contexts [30,31,33,35], their cellular and soluble profiles in children with CL/P remain insufficiently characterized. Our observations therefore provide preliminary information that may support further investigations into immune-regulatory phenotypes in this population.

Subgroup analyses by sex and mode of delivery showed limited evidence of consistent differences in immune checkpoint-related parameters. We found no statistically significant differences between boys and girls in the soluble markers or checkpoint-positive lymphocyte populations investigated. Although sex-related differences have been described in the epidemiology of orofacial clefts [1], our findings did not show a clear sex-associated pattern in the immunological parameters analyzed. Regarding delivery mode, the proportion of CD19+ PD-L1+ B lymphocytes was higher among children born by cesarean section than among those delivered vaginally. Previous research has suggested that delivery mode may influence early microbial colonization and immune development [38]. Nevertheless, we should interpret the isolated association observed in our study cautiously because of the relatively small subgroup sizes and the exploratory nature of multiple statistical comparisons. Stratification by delivery mode also revealed differences in the direction or strength of selected correlations, particularly those involving birth weight and IgG concentrations and the relationship between sPD-1 and sCTLA-4. However, these observations do not establish a direct influence of delivery mode on immune checkpoint regulation, and the clinical significance of the subgroup-specific correlations remains uncertain.

The ROC analyses showed exceptionally high discriminatory performance for the immune checkpoint-related parameters investigated, with AUC values of 1.00 for all three soluble markers and 0.92 to 1.00 for the cellular markers. These findings may be partly explained by the pronounced differences in immune checkpoint-related parameters observed between children with CL/P and healthy controls in the present cohort. However, an AUC of 1.00 indicates complete discrimination within the analyzed sample and should not be interpreted as evidence of perfect diagnostic performance in the broader pediatric population. The relatively small sample size, particularly in the control group, may affect the stability of these estimates and limit their generalizability. Furthermore, differences in clinical and demographic characteristics between the investigated groups, as well as potential unmeasured confounding factors, cannot be excluded as contributors to the observed discriminatory performance. Importantly, the ROC analyses were performed within the original study cohort without validation in an independent population. Therefore, these findings should be regarded as exploratory measures of within-sample discrimination rather than evidence of established diagnostic utility. Confirmation in larger, independently recruited cohorts, with appropriate consideration of potential confounding factors, is necessary to determine the reproducibility and clinical relevance of these results.

Several limitations should be acknowledged when interpreting the present findings. First, this single-center study included a relatively small cohort of 32 children with CL/P and 16 healthy controls. The limited sample size, particularly in the control group, reduces the precision of effect estimates and the statistical power of subgroup comparisons, while increasing the susceptibility of correlation analyses to sampling variability. Moreover, recruitment from a single medical center may limit the representativeness of the study population and the generalizability of the findings to children with different clinical characteristics and cleft phenotypes. Although the investigated groups were comparable in age and sex distribution, we observed differences in selected perinatal characteristics, particularly birth weight, preterm birth frequency, and mode of delivery. These factors may contribute to variability in immune phenotypes and may have confounded the results, as we could not adequately control for them in the present study. Therefore, we should not attribute the observed immunological differences exclusively to CL/P.

Another important limitation is the cross-sectional design, which involved a single assessment of immune checkpoint-related parameters. This approach was selected to provide an initial characterization of immune checkpoint profiles in a clinically accessible pediatric cohort. Because eligible patients were limited to a single medical center, we did not conduct repeated blood sampling or longitudinal follow-up. Consequently, we were unable to determine whether the observed differences in cellular PD-1, PD-L1, and CTLA-4 expression and their soluble counterparts represent persistent immunological characteristics of children with CL/P or transient changes associated with previous inflammatory or infectious exposures. Although we recorded age and excluded active infections at enrollment, we did not systematically assess detailed infection histories or the potential influence of prior surgical interventions. Furthermore, the study design does not allow us to establish temporal or causal relationships between immune checkpoint-related parameters and the development or clinical manifestations of CL/P. Future prospective studies incorporating repeated immune assessments, documented infectious episodes, and surgical histories are needed to establish the temporal stability and potential clinical relevance of these findings.

Our investigation phenotypically characterized PD-1, PD-L1, and CTLA-4 expression in selected T- and B-lymphocyte populations and measured their soluble forms in serum. Although these parameters differed significantly between children with CL/P and healthy controls, increased expression of immune checkpoint molecules does not necessarily indicate impaired lymphocyte function, immune suppression, or cellular exhaustion. These molecules may also reflect physiological immune activation, antigenic stimulation, or regulatory processes. Similarly, elevated concentrations of soluble immune checkpoint molecules cannot be directly interpreted as evidence of altered checkpoint signaling activity. Therefore, the functional significance of the observed immunophenotypic differences remains uncertain. Further studies incorporating ex vivo lymphocyte stimulation, proliferation assays, cytokine production measurements, and functional evaluation of PD-1/PD-L1- and CTLA-4-related pathways would help determine whether the identified phenotypic alterations are associated with measurable changes in immune cell function.

An additional consideration is the complex genetic background of CL/P, which is a multifactorial condition involving numerous susceptibility genes and genetic variants, including those associated with *IRF6*, *PAX7*, and *VAX1* [39,40]. This genetic heterogeneity may also contribute to interindividual variability in immune phenotypes, independently of or in combination with postnatal environmental and clinical factors. However, the present study did not include genetic analyses, and therefore, we cannot determine whether the observed differences in cellular and soluble immune checkpoint-related parameters are associated with the underlying genetic background of CL/P. Importantly, our findings do not establish a direct relationship between CL/P-associated genetic variants and the regulation of PD-1, PD-L1, or CTLA-4. Future studies integrating genetic characterization with detailed immunophenotyping may help clarify the potential contribution of genetic factors to immune-regulatory profiles in children with CL/P.

Despite these limitations, the present study provides preliminary evidence of differences in cellular and soluble immune checkpoint-related parameters in children with CL/P compared with healthy controls. These findings expand the descriptive data on immune phenotypes in this population and highlight the need for larger, prospective, multicenter studies that integrate longitudinal, functional, and genetic assessments. Such investigations are necessary to determine whether the observed immunological differences are reproducible and to clarify their potential biological and clinical significance.

## 4. Materials and Methods

### 4.1. Characteristics of the Patient Population

This exploratory, single-center, cross-sectional study characterized cellular and soluble immune checkpoint-related parameters in children with CL/P compared with healthy pediatric controls. A single peripheral blood sample was obtained from each participant. We used a cross-sectional approach to provide an initial assessment of immune checkpoint profiles within the practical constraints of recruiting eligible pediatric participants at a single medical center. We did not perform longitudinal sampling, and the study was not intended to establish temporal or causal relationships between immune checkpoint-related parameters and CL/P clinical characteristics. A total of 48 pediatric participants were enrolled: 32 with a diagnosis of CL/P (study group) and 16 healthy volunteers (control group). We applied the same inclusion and exclusion criteria to both groups.

The study group comprised 32 patients aged 2–17 years (mean age, 9.44 years), with various cleft phenotypes. Fifteen children (46.87%) had an isolated cleft of the soft or hard palate, six (18.75%) had a combined soft and hard palate cleft, six (18.75%) had a cleft lip, and five (15.63%) had a cleft lip with a cleft palate. The group included 17 boys and 15 girls. Regarding perinatal factors, 19 children (59.38%) were delivered by cesarean section and 13 (40.62%) vaginally; four patients (12.5%) were born prematurely.

The control group consisted of 16 healthy pediatric volunteers without craniofacial malformations or a history of chronic immune-mediated disease. The mean age in the control group was 9.81 years (range 4–16 years); nine children were male, and seven were female. Four children (25%) were born by cesarean section and 12 (75%) vaginally; seven participants (43.75%) were born prematurely.

Participants were eligible if they were <18 years old, had not received immunosuppressive therapy within 12 months before enrollment, had not received antibiotic treatment within 3 months before blood sampling, and provided written consent from a parent/legal guardian (and assent from the child, when applicable). Exclusion criteria included active viral, bacterial, or fungal infection at enrollment; diabetes or other relevant metabolic/endocrine disorders; history of hematopoietic stem cell or solid organ transplantation; active malignancy or autoimmune disease; pregnancy or lactation; use of medications affecting immune function (including investigational drugs); and severe mental disorders preventing cooperation.

We collected peripheral venous blood in the morning in an outpatient setting. Blood was drawn into EDTA tubes for complete blood count and flow cytometric immunophenotyping and into serum tubes to assess soluble immune checkpoint molecules.

To assess baseline comparability between the study and control groups, we evaluated demographic, anthropometric, and perinatal characteristics, including age, sex, height, body weight, birth weight, mode of delivery, and preterm birth status. Continuous variables were summarized as mean ± standard deviation (SD) and median with interquartile range (Q1–Q3), whereas categorical variables were presented as numbers and percentages. Table 10 summarizes the baseline characteristics of both groups and the corresponding statistical comparisons.

Peripheral venous blood samples were collected in the morning during outpatient visits. Participants with active viral, bacterial, or fungal infections at the time of enrollment were excluded from the study. Blood was collected into EDTA tubes for complete blood count and flow cytometric immunophenotyping and into serum collection tubes for the assessment of soluble immune checkpoint molecules. We performed sample collection, processing, and laboratory analyses according to standardized procedures. Additional information regarding preanalytical conditions, sample processing, storage, and assay organization is provided in Supplementary Methods S1.

### 4.2. Flow Cytometric Immunophenotyping

We performed peripheral blood immunophenotyping using multiparameter flow cytometry to determine the distribution of major lymphocyte populations and the surface expression of selected immune checkpoint molecules. Fresh peripheral blood samples were stained with fluorochrome-conjugated monoclonal antibodies directed against lineage-defining markers, including anti-CD45 Alexa Fluor 700, anti-CD3 PerCP, anti-CD4 BV421, anti-CD8 BV605, and anti-CD19 FITC, as well as antibodies targeting the immune checkpoint molecules PD-1 (CD279), PD-L1 (CD274), and CTLA-4 (CD152). Anti-PD-1 APC, anti-PD-L1 PE, and anti-CTLA-4 PE antibodies were used for checkpoint assessment. All monoclonal antibodies were obtained from BioLegend (San Diego, CA, USA).

Following antibody incubation, erythrocytes were lysed using a dedicated lysing buffer (BD Biosciences, Franklin Lakes, NJ, USA), and the remaining cells were washed prior to cytometric acquisition. Samples were analyzed using a CytoFLEX LX flow cytometer (Beckman Coulter, Indianapolis, IN, USA). Instrument performance was monitored by routine quality-control procedures using CytoFLEX Ready-to-Use Daily QC Fluorospheres (Beckman Coulter) before sample acquisition. Fluorescence-minus-one (FMO) controls were included for immune checkpoint markers to support consistent definition of positive and negative cell populations.

The gating strategy was applied uniformly to all samples. After excluding debris and doublets, we identified the lymphocyte population based on forward- and side-scatter characteristics. We identified T lymphocytes as CD3+ cells and further subdivided them into CD3+ CD4+ and CD3+ CD8+ populations, whereas we identified B lymphocytes as CD19+ cells. Within each defined lymphocyte population, the proportions of cells positive for PD-1 (CD279), PD-L1 (CD274), and CTLA-4 (CD152) were determined. Results were expressed as the percentage of checkpoint-positive cells within the respective parent lymphocyte population. Flow cytometric data were analyzed using Kaluza Analysis software version 2.1 (Beckman Coulter), with the same gating strategy and positivity thresholds applied throughout the study.

### 4.3. Assessment of Soluble Immune Checkpoint Molecules

Serum concentrations of soluble PD-1 (sPD-1), soluble PD-L1 (sPD-L1), and soluble CTLA-4 (sCTLA-4) were determined using commercially available enzyme-linked immunosorbent assay (ELISA) kits. Serum samples collected from study participants were processed and stored under standardized conditions until analysis. We performed all assays according to the manufacturers’ instructions, using the same analytical procedure for samples from children with CL/P and healthy controls.

Commercial ELISA kits for human PD-1 (CD279), PD-L1 (CD274), and CTLA-4 (CD152) were obtained from Invitrogen (Waltham, MA, USA). The reported analytical sensitivities of the assays were 1.14 pg/mL for sPD-1, 0.6 pg/mL for sPD-L1, and 0.13 ng/mL for sCTLA-4. We prepared standards according to the manufacturers’ protocols and serially diluted them to generate individual calibration curves for each analyte. Serum samples and standards underwent sequential incubation, washing, detection-antibody, and substrate-development steps as specified for each assay.

Absorbance was measured at 450 nm using a Victor 3 microplate reader (PerkinElmer, Waltham, MA, USA), with reference wavelength correction within the 570–620 nm range when required by the assay protocol. Analyte concentrations were calculated from the corresponding standard curves and expressed in the units specified for each assay.

### 4.4. Statistical Analysis

Statistical analyses were performed using GraphPad Prism version 10.6.1 (GraphPad Software, LLC, Boston, MA, USA) and Statistica version 14 (TIBCO Software Inc., Palo Alto, CA, USA). Because most variables were not normally distributed, we used nonparametric methods. Comparisons between two independent groups (study vs. control; boys vs. girls; vaginal delivery vs. cesarean section) were conducted using the Mann–Whitney U test. *p* values < 0.05 were considered statistically significant. We compared categorical demographic and perinatal variables between the study and control groups using Fisher’s exact test. We evaluated associations between clinical, hematological, and immunological variables using Spearman’s rank correlation coefficient (ρ). For each correlation, the correlation coefficient (r/ρ), the corresponding t statistic (*n* − 2), and the *p* value were reported. Results were summarized in tables and visualized using heat maps reflecting the strength and direction of correlations. We performed Receiver Operating Characteristic (ROC) curve analysis to evaluate the discriminatory performance of the investigated immune checkpoint parameters. The area under the curve (AUC), 95% confidence intervals, standard errors, and *p*-values for AUC significance tests were calculated. We conducted ROC analyses to assess the ability of the studied markers to differentiate children with CL/P from controls and to explore subgroup differences by sex and delivery mode. AUC interpretation followed commonly used thresholds: 0.5–0.6 (no discrimination), 0.6–0.75 (poor), 0.75–0.9 (good), 0.9–1.0 (excellent), and 1.0 (perfect discrimination). Descriptive data are presented as median and interquartile range (Q1–Q3).

## 5. Conclusions

In the group of children with CL/P, the correlation profile was dominated by strong physiological developmental and hematological associations (age, height, weight, leukocytes, neutrophils, erythrocytes, and hemoglobin). We also demonstrated co-regulation of immune checkpoints in the PD-1/PD-L1 and CTLA-4 axis, including correlations of soluble markers and associations between T and B lymphocyte phenotypes. Key correlations were similar in boys and girls, suggesting a stable pattern of association regardless of gender, whereas stratification by delivery type revealed heterogeneity in selected relationships (a different direction of the relationship between birth weight and IgG and a stronger correlation between sPD-1 and sCTLA-4 after cesarean section). Because of the small sample size, single-center design, cross-sectional design, and lack of functional and genetic analyses, the results should be considered hypothesis-generating and require confirmation in larger, multi-center studies.

## Figures and Tables

**Table 1:** Comparison of immunoglobulins, peripheral blood morphology parameters, and immunophenotype (flow cytometry) in children with CL/P (*n* = 32) and in healthy children (*n* = 16).

Parameter	Study Group (*n* = 32)		Control Group (*n* = 16)		*p*-Value
	Mean ± SD	Median (Q1–Q3)	Mean ± SD	Median (Q1–Q3)	
Immunoglobulins					
IgG (mg/dL)	1091.34 ± 151.90	1077.50 (996.25–1175.00)	949.73 ± 307.11	980.05 (919.63–1110.75)	0.0852
IgA (mg/dL)	132.94 ± 54.59	129.00 (101.00–166.50)	173.95 ± 58.23	172.70 (149.23–190.28)	0.0103
IgM (mg/dL)	105.47 ± 47.60	97.00 (74.75–135.75)	114.00 ± 39.16	119.90 (95.95–132.65)	0.3480
IgE (mg/dL)	142.01 ± 237.32	52.60 (16.60–141.10)	228.21 ± 130.99	207.45 (159.20–358.53)	0.0068
Complete Blood Count—Erythrocyte Indices					
Red blood cells (mL/µL)	4.73 ± 0.34	4.78 (4.53–4.94)	3.93 ± 0.68	3.80 (3.40–4.67)	0.0004
Hemoglobin (g/dL)	12.85 ± 1.02	12.85 (11.98–13.50)	11.47 ± 0.89	11.21 (10.86–11.95)	<0.0001
Complete Blood Count—Platelets					
Platelets (×10^3^/µL)	288.63 ± 59.25	276.50 (253.25–319.75)	283.75 ± 74.41	296.00 (232.25–360.00)	0.8077
Complete Blood Count—White Blood Cells					
White blood cells (×10^3^/µL)	7.57 ± 2.06	7.07 (5.87–9.13)	8.46 ± 2.39	9.42 (7.14–10.13)	0.1458
Lymphocytes (×10^3^/mL)	2.87 ± 0.93	2.68 (2.31–3.03)	2.93 ± 0.73	2.89 (2.47–3.47)	0.5052
Neutrophils (×10^3^/mL)	3.80 ± 1.77	3.44 (2.40–5.20)	4.70 ± 1.56	5.10 (3.61–5.96)	0.0445
Monocytes (×10^3^/µL)	0.61 ± 0.16	0.55 (0.46–0.72)	0.46 ± 0.15	0.47 (0.39–0.53)	0.0089
Immunophenotyping					
CD45+ leukocytes (%)	83.09 ± 10.27	85.84 (76.86–90.34)	96.35 ± 2.41	97.38 (95.45–98.09)	<0.0001
CD3+ T lymphocytes (%)	59.86 ± 8.70	59.50 (52.83–66.49)	73.12 ± 3.18	72.34 (71.21–75.32)	<0.0001
CD3+ CD4+ T lymphocytes (%)	27.30 ± 6.10	26.41 (23.01–31.33)	42.88 ± 3.44	43.70 (40.71–45.74)	<0.0001
CD3+ CD8+ T lymphocytes (%)	32.56 ± 7.75	32.61 (27.45–36.42)	30.25 ± 4.04	29.27 (28.01–32.11)	0.3233
CD19+ B lymphocytes (%)	8.09 ± 2.02	7.68 (6.61–9.93)	12.24 ± 2.61	12.17 (11.19–13.46)	<0.0001

**Table 2:** Comparison of the percentage of CD4+, CD8+, and CD19+ lymphocytes expressing PD-1, PD-L1, and CTLA-4 and serum levels of sPD-1, sPD-L1, and sCTLA-4 between children with CL/P (*n* = 32) and healthy controls (*n* = 16).

Parameter	Study Group (*n* = 32)		Control Group (*n* = 16)		*p*-Value
	Mean ± SD	Median (Q1–Q3)	Mean ± SD	Median (Q1–Q3)	
CD4+ T cells					
CD4+ PD-1+ (%)	4.58 ± 1.60	4.61 (3.39–5.74)	0.85 ± 0.56	0.75 (0.52–0.89)	<0.0001
CD4+ PD-L1+ (%)	9.01 ± 3.31	9.46 (7.14–11.67)	0.85 ± 0.55	0.70 (0.57–0.93)	<0.0001
CD4+ CTLA-4+ (%)	10.83 ± 4.47	9.76 (7.38–14.51)	0.96 ± 0.48	0.91 (0.62–1.07)	<0.0001
CD8+ T cells					
CD8+ PD-1+ (%)	16.19 ± 4.64	16.67 (12.86–20.05)	0.76 ± 0.67	0.45 (0.39–0.85)	<0.0001
CD8+ PD-L1+ (%)	8.18 ± 4.36	9.25 (4.41–11.94)	0.86 ± 0.70	0.53 (0.35–1.38)	<0.0001
CD8+ CTLA-4+ (%)	14.48 ± 4.34	14.82 (10.92–17.67)	1.00 ± 0.79	0.59 (0.40–1.61)	<0.0001
CD19+ B cells					
CD19+ PD-1+ (%)	5.16 ± 1.80	5.61 (3.65–6.68)	2.29 ± 0.69	2.40 (1.90–2.56)	<0.0001
CD19+ PD-L1+ (%)	15.06 ± 8.96	15.19 (8.03–23.06)	1.41 ± 0.45	1.34 (1.12–1.61)	<0.0001
CD19+ CTLA-4+ (%)	10.96 ± 6.05	12.72 (5.32–16.01)	1.02 ± 0.25	1.07 (0.75–1.18)	<0.0001
Soluble markers (serum)					
sPD-1 (ng/mL)	24.95 ± 4.03	24.80 (22.24–28.07)	4.40 ± 0.75	4.48 (3.66–4.99)	<0.0001
sPD-L1 (ng/mL)	34.37 ± 4.42	34.14 (31.00–36.92)	4.20 ± 0.91	3.93 (3.41–4.89)	<0.0001
sCTLA-4 (ng/mL)	41.11 ± 4.55	41.02 (38.16–44.46)	5.09 ± 1.09	4.84 (4.16–6.17)	<0.0001

**Table 3:** Comparison of soluble markers (sPD-1, sPD-L1, sCTLA-4) and percentages of CD4+, CD8+, and CD19+ lymphocytes expressing PD-1, PD-L1, and CTLA-4 depending on gender in the group of patients with CL/P (male *n* = 17; female *n* = 15).

Parameter	Male (*n* = 17)		Female (*n* = 15)		*p*-Value
	Mean ± SD	Median (Q1–Q3)	Mean ± SD	Median (Q1–Q3)	
Soluble markers (serum)					
sPD-1 (ng/mL)	25.70 ± 4.25	26.30 (22.38–29.33)	24.11 ± 3.58	24.38 (21.97–25.53)	0.1890
sPD-L1 (ng/mL)	35.79 ± 3.95	35.55 (33.83–38.28)	32.77 ± 4.38	32.03 (29.77–35.92)	0.0651
sCTLA-4 (ng/mL)	41.73 ± 3.99	41.92 (39.30–44.16)	40.40 ± 5.02	40.13 (38.01–44.17)	0.4784
CD4+ T cells					
CD4+ PD-1+ (%)	4.96 ± 1.38	4.99 (3.75–5.74)	4.14 ± 1.71	4.54 (2.56–5.64)	0.2416
CD4+ PD-L1+ (%)	8.63 ± 3.63	8.40 (6.41–12.02)	9.44 ± 2.85	9.78 (7.96–11.10)	0.6281
CD4+ CTLA-4+ (%)	10.93 ± 4.27	10.70 (7.09–14.43)	10.71 ± 4.69	9.31 (7.64–12.52)	0.8523
CD8+ T cells					
CD8+ PD-1+ (%)	15.48 ± 5.21	14.92 (10.47–20.05)	16.98 ± 3.74	17.44 (14.29–19.49)	0.4613
CD8+ PD-L1+ (%)	9.37 ± 3.71	9.74 (7.37–12.45)	6.84 ± 4.65	5.36 (2.05–11.38)	0.0894
CD8+ CTLA-4+ (%)	15.50 ± 3.15	15.56 (13.26–17.60)	13.33 ± 5.14	11.54 (9.70–17.87)	0.1645
CD19+ B cells					
CD19+ PD-1+ (%)	5.14 ± 1.93	5.53 (3.16–6.85)	5.17 ± 1.63	5.80 (3.83–6.51)	>0.9999
CD19+ PD-L1+ (%)	15.97 ± 10.09	19.11 (5.76–26.30)	14.03 ± 7.36	12.05 (9.49–20.84)	0.2948
CD19+ CTLA-4+ (%)	9.56 ± 6.46	7.32 (3.31–15.70)	12.55 ± 5.10	13.91 (7.42–17.32)	0.1425

**Table 4:** Comparison of PD-1/PD-L1/CTLA-4 expression on CD4+, CD8+, and CD19+ lymphocytes and sPD-1, sPD-L1, and sCTLA-4 concentrations depending on the type of delivery (natural delivery vs. cesarean section) in the study group.

Parameter	N—Vaginal Delivery (*n* = 13)		CC—Cesarean Section (*n* = 19)		*p*-Value
	Mean ± SD	Median (Q1–Q3)	Mean ± SD	Median (Q1–Q3)	
Soluble markers (serum)					
sPD-1 (ng/mL)	24.37 ± 4.08	22.53 (21.41–26.30)	25.35 ± 3.95	25.64 (23.10–28.11)	0.3234
sPD-L1 (ng/mL)	33.92 ± 4.48	34.30 (30.47–36.82)	34.69 ± 4.35	34.14 (31.59–36.99)	0.6982
sCTLA-4 (ng/mL)	40.24 ± 4.62	40.78 (37.23–42.20)	41.70 ± 4.40	41.27 (38.36–45.75)	0.5701
CD4+ T cells					
CD4+ PD-1+ (%)	4.41 ± 1.82	3.75 (3.43–5.46)	4.69 ± 1.41	4.99 (3.84–5.74)	0.5258
CD4+ PD-L1+ (%)	9.38 ± 2.91	9.78 (7.46–11.49)	8.75 ± 3.53	8.47 (6.53–11.88)	0.7623
CD4+ CTLA-4+ (%)	10.63 ± 5.22	8.60 (6.78–16.95)	10.96 ± 3.88	10.08 (7.64–14.29)	0.6498
CD8+ T cells					
CD8+ PD-1+ (%)	15.24 ± 4.61	14.69 (12.61–17.44)	16.83 ± 4.56	18.45 (14.48–20.21)	0.2343
CD8+ PD-L1+ (%)	8.67 ± 4.29	10.07 (5.36–11.96)	7.85 ± 4.38	8.77 (3.42–11.66)	0.5446
CD8+ CTLA-4+ (%)	14.14 ± 3.84	14.76 (10.86–16.97)	14.72 ± 4.63	14.88 (11.70–17.75)	0.7336
CD19+ B cells					
CD19+ PD-1+ (%)	5.08 ± 1.96	6.17 (3.16–6.36)	5.21 ± 1.67	5.53 (4.12–6.77)	0.9698
CD19+ PD-L1+ (%)	11.21 ± 8.93	8.50 (1.83–19.11)	21.97 ± 12.02	23.52 (17.69–27.98)	0.0493
CD19+ CTLA-4+ (%)	12.66 ± 5.93	13.91 (7.32–17.61)	9.80 ± 5.85	11.41 (3.87–14.84)	0.1361

**Table 5:** Comparison of correlation coefficients r between boys and girls in the cleft group.

Variable Pair	Male		Female	
	r	*p*	r	*p*
Age (years) and Body weight (kg)	0.850	0.000	0.878	0.000
Age (years) and Height (cm)	0.892	0.000	0.965	0.000
Height (cm) and Body weight (kg)	0.919	0.000	0.930	0.000
White blood cells (×10^3^/µL) and Neutrophils (×10^3^/µL)	0.902	0.000	0.864	0.000
Red blood cells (×10^6^/µL) and Hemoglobin (g/dL)	0.703	0.002	0.660	0.007
CD4+ PD-L1+ and CD4+ CTLA-4+	0.583	0.014	0.657	0.008

**Table 6:** Comparison of significant correlations (r) depending on the type of delivery in the group with cleft palate.

Variable Pair	N—Vaginal Delivery		CC—Cesarean Section	
	r	*p*	r	*p*
Birth weight (g) and IgG (mg/dL)	−0.606	0.048	0.470	0.042
CD19+ and CD8+ PD-L1+	−0.588	0.035	−0.495	0.031
sPD-1 and sCTLA-4	0.555	0.049	0.711	0.001
Red blood cells (×10^6^/µL) and Hemoglobin (g/dL)	0.798	0.001	0.744	0.000
CD3+ and CD8+	0.725	0.005	0.781	0.000
Age (years) and Body weight (kg)	0.785	0.002	0.821	0.000
Age (years) and Height (cm)	0.865	0.000	0.825	0.000
Height (cm) and Body weight (kg)	0.954	0.000	0.937	0.000
White blood cells (×10^3^/µL) and Neutrophils (×10^3^/µL)	0.962	0.000	0.882	0.000

**Table 7:** ROC analysis (AUC) for soluble markers (sPD-1, sPD-L1, sCTLA-4) and PD-1/PD-L1/CTLA-4 expression on CD4+, CD8+ and CD19+ lymphocytes in differentiating children with CP/L from the control group.

Marker	AUC	SE	95% CI	*p*
Soluble markers (serum)				
sPD-1 (ng/mL)	1.000	0.000	1.000–1.000	<0.0001
sPD-L1 (ng/mL)	1.000	0.000	1.000–1.000	<0.0001
sCTLA-4 (ng/mL)	1.000	0.000	1.000–1.000	<0.0001
CD4+ T cells				
CD4+ PD-1+ (%)	0.992	0.009	0.974–1.000	<0.0001
CD4+ PD-L1+ (%)	0.994	0.007	0.980–1.000	<0.0001
CD4+ CTLA-4+ (%)	1.000	0.000	1.000–1.000	<0.0001
CD8+ T cells				
CD8+ PD-1+ (%)	1.000	0.000	1.000–1.000	<0.0001
CD8+ PD-L1+ (%)	0.956	0.026	0.906–1.000	<0.0001
CD8+ CTLA-4+ (%)	1.000	0.000	1.000–1.000	<0.0001
CD19+ B cells				
CD19+ PD-1+ (%)	0.920	0.037	0.847–0.993	<0.0001
CD19+ PD-L1+ (%)	0.928	0.036	0.858–0.998	<0.0001

**Table 8:** ROC analysis (AUC) for soluble and cellular markers in predicting gender (boys vs. girls) in the cleft group.

Marker	AUC	SE	95% CI	*p*
Soluble markers (serum)				
sPD-1 (ng/mL)	0.639	0.102	0.440–0.839	0.180
sPD-L1 (ng/mL)	0.692	0.096	0.505–0.879	0.064
sCTLA-4 (ng/mL)	0.577	0.105	0.371–0.782	0.462
CD4+ T cells				
CD4+ PD-1+ (%)	0.624	0.104	0.419–0.828	0.234
CD4+ PD-L1+ (%)	0.553	0.105	0.347–0.759	0.610
CD4+ CTLA-4+ (%)	0.522	0.106	0.313–0.730	0.836
CD8+ T cells				
CD8+ PD-1+ (%)	0.578	0.103	0.376–0.781	0.450
CD8+ PD-L1+ (%)	0.678	0.097	0.488–0.869	0.086
CD8+ CTLA-4+ (%)	0.647	0.108	0.435–0.859	0.157
CD19+ B cells				
CD19+ PD-1+ (%)	0.502	0.104	0.298–0.706	0.985
CD19+ PD-L1+ (%)	0.612	0.103	0.410–0.814	0.282
CD19+ CTLA-4+ (%)	0.655	0.098	0.462–0.848	0.136

**Table 9:** ROC analysis (AUC) for soluble and cellular markers in predicting the type of delivery (vaginal delivery vs. cesarean section) in the cleft group.

Marker	AUC	SE	95% CI	*p*
Soluble markers (serum)				
sPD-1 (ng/mL)	0.607	0.107	0.398–0.817	0.309
sPD-L1 (ng/mL)	0.543	0.108	0.332–0.753	0.687
sCTLA-4 (ng/mL)	0.563	0.104	0.358–0.767	0.552
CD4+ T cells				
CD4+ PD-1+ (%)	0.569	0.110	0.353–0.785	0.514
CD4+ PD-L1+ (%)	0.534	0.105	0.329–0.740	0.744
CD4+ CTLA-4+ (%)	0.539	0.104	0.334–0.743	0.716
CD8+ T cells				
CD8+ PD-1+ (%)	0.628	0.108	0.415–0.840	0.227
CD8+ PD-L1+ (%)	0.567	0.105	0.360–0.773	0.527
CD8+ CTLA-4+ (%)	0.551	0.116	0.324–0.777	0.632
CD19+ B cells				
CD19+ PD-1+ (%)	0.506	0.110	0.290–0.722	0.954
CD19+ PD-L1+ (%)	0.709	0.099	0.515–0.902	0.048
CD19+ CTLA-4+ (%)	0.660	0.102	0.461–0.859	0.130

**Table 10:** Baseline clinical and anthropometric characteristics of the study and control groups.

Parameter	CL/P Group (*n* = 32)	Control Group (*n* = 16)	*p*-Value
Demographic characteristics			
Age (years), mean ± SD	9.44 ± 4.23	9.81 ± 3.56	0.7078
Age, median (Q1–Q3)	8.50 (6.00–12.50)	9.50 (6.75–12.25)	—
Male sex, *n* (%)	17 (53.1%)	9 (56.3%)	1.0000
Female sex, *n* (%)	15 (46.9%)	7 (43.8%)	—
Anthropometric characteristics			
Height (cm), mean ± SD	137.66 ± 22.44	141.46 ± 20.39	0.5843
Height, median (Q1–Q3)	137.00 (119.25–157.00)	141.95 (122.93–155.05)	—
Body weight (kg), mean ± SD	31.30 ± 15.70	33.98 ± 16.63	0.9682
Body weight, median (Q1–Q3)	29.00 (21.25–39.75)	29.15 (22.25–35.50)	—
Perinatal characteristics			
Birth weight (g), mean ± SD	3170.78 ± 439.98	3018.81 ± 576.26	0.0397
Birth weight, median (Q1–Q3)	3380.00 (3062.50–3665.00)	3039.00 (2441.25–3499.50)	—
Vaginal delivery, *n* (%)	13 (40.6%)	12 (75.0%)	—
Cesarean section, *n* (%)	19 (59.4%)	4 (25.0%)	0.0339
Preterm birth, *n* (%)	4 (12.5%)	7 (43.8%)	0.0270
Term birth, *n* (%)	28 (87.5%)	9 (56.3%)	—

Data are presented as mean ± standard deviation (SD), median (first quartile [Q1]–third quartile [Q3]), or number (percentage), as appropriate. We compared continuous variables using the Mann–Whitney U test and categorical variables using Fisher’s exact test. For binary categorical variables, one *p*-value is reported per comparison. We considered *p*-values less than 0.05 statistically significant.

## Data Availability

The data supporting the findings of this study are available from the first author upon reasonable written request.

## Supplementary Materials

The following supporting information can be downloaded at: https://www.mdpi.com/article/10.3390/ijms27219488/s1.
