# Supplementary material for: Checkpoint Pathways in Childhood Cleft Lip and Palate—Associations of PD1 and CTLA4 with Clinical Parameters
Source: Int J Mol Sci. 2026 Oct 24;27(21):9488. doi: 10.3390/ijms27219488 (PMC13762904; doi:10.3390/ijms27219488)
Supplement: Supplementary file 1 [file ijms-27-09488-s001.zip › ijms-4608055-supplementary.pdf]

Supplementary materials

Table S1. Significant pairwise correlations between immune checkpoint parameters and clinical/laboratory variables in children with CL/P (study group).

Variable pair	r	t (N−2)	p
CD19+PD-L1+ & CD19+CTLA-4+	-0.777	-6.755	0.000
Age (years) & Lymphocytes (×10³/mL)	-0.525	-3.376	0.002
CD19+ (%) & CD8+PD-L1+ (%)	-0.474	-2.951	0.006
Hemoglobin (g/dL) & Monocytes (×10³/µL)	-0.456	-2.805	0.009
Height (cm) & Lymphocytes (×10³/mL)	-0.433	-2.634	0.013
Body weight (kg) & Lymphocytes (×10³/mL)	-0.424	-2.480	0.019
CD4+PD-L1+ (%) & CD19+PD-1+ (%)	-0.405	-2.427	0.021
Body weight (kg) & White blood cells (×10³/µL)	-0.402	-2.324	0.028
CD4+CTLA-4+ (%) & CD19+CTLA-4+ (%)	-0.379	-2.241	0.033
sPD-L1 (serum) & CD8+CTLA-4+ (%)	0.350	2.049	0.049
Monocytes (×10³/µL) & sPD-L1 (serum)	0.350	2.050	0.049
Age (years) & IgG (mg/dL)	0.352	2.059	0.048
IgG (mg/dL) & sCTLA-4 (serum)	0.352	2.063	0.048
CD4+CTLA-4+ (%) & CD19+PD-L1+ (%)	0.362	2.128	0.042
IgA (mg/dL) & IgG (mg/dL)	0.362	2.094	0.045
Platelets (×10³/µL) & CD4+ (%)	0.364	2.142	0.040
IgM (mg/dL) & IgG (mg/dL)	0.368	2.167	0.038
IgM (mg/dL) & IgE (mg/dL)	0.369	2.173	0.038
CD3+ (%) & sPD-1 (serum)	0.379	2.243	0.032
Body weight (kg) & CD19+CTLA-4+ (%)	0.380	2.173	0.038
sCTLA-4 (serum) & CD8+CTLA-4+ (%)	0.381	2.259	0.031
IgM (mg/dL) & CD19+CTLA-4+ (%)	0.384	2.281	0.030
sPD-L1 (serum) & CD19+PD-L1+ (%)	0.394	2.351	0.025
Platelets (×10³/µL) & CD8+CTLA-4+ (%)	0.396	2.365	0.025
sCTLA-4 (serum) & CD19+PD-L1+ (%)	0.408	2.448	0.020
Body weight (kg) & Birth weight (g)	0.411	2.343	0.027
sPD-1 (serum) & CD8+PD-L1+ (%)	0.413	2.485	0.019
Red blood cells (×10⁶/µL) & CD8+PD-1+ (%)	0.413	2.486	0.019
Height (cm) & Hemoglobin (g/dL)	0.418	2.518	0.017
CD8+ (%) & CD8+PD-1+ (%)	0.424	2.562	0.016
IgA (mg/dL) & Monocytes (×10³/µL)	0.438	2.621	0.014
CD3+ (%) & CD8+PD-1+ (%)	0.440	2.683	0.012
Age (years) & Hemoglobin (g/dL)	0.453	2.780	0.009
Hemoglobin (g/dL) & CD8+PD-1+ (%)	0.463	2.860	0.008
CD3+ (%) & CD4+ (%)	0.464	2.870	0.007
Birth weight (g) & IgA (mg/dL)	0.465	2.731	0.011
sPD-1 (serum) & CD8+CTLA-4+ (%)	0.469	2.907	0.007
Neutrophils (×10³/mL) & Monocytes (×10³/µL)	0.475	2.958	0.006
CD8+PD-1+ (%) & CD19+CTLA-4+ (%)	0.477	2.975	0.006
CD45+ (%) & CD8+ (%)	0.519	3.326	0.002
CD4+PD-L1+ (%) & CD4+CTLA-4+ (%)	0.559	3.693	0.001
CD45+ (%) & CD3+ (%)	0.606	4.168	0.000
sPD-1 (serum) & sCTLA-4 (serum)	0.638	4.540	0.000

White blood cells (×10^3/μL) & Monocytes (×10^3/μL)	0.650	4.681	0.000
Red blood cells (×10^6/μL) & Hemoglobin (g/dL)	0.690	5.221	0.000
CD3+ (%) & CD8+ (%)	0.772	6.652	0.000
Age (years) & Body weight (kg)	0.835	8.016	0.000
White blood cells (×10^3/μL) & Neutrophils (×10^3/mL)	0.891	10.751	0.000
Age (years) & Height (cm)	0.915	12.443	0.000
Height (cm) & Body weight (kg)	0.926	12.944	0.000

Table S2. Significant correlations in boys with CL/P (study group)

**Variable pair**	**r**	**t (N–2)**	**p-value**
CD4+PD-L1+ [%] & CD19+PD-1+ [%]	-0.593	-2.853	0.012
Age (years) & Platelets (×10^3/μL)	-0.591	-2.836	0.013
Height (cm) & Platelets (×10^3/μL)	-0.568	-2.672	0.017
Body weight (kg) & Platelets (×10^3/μL)	-0.561	-2.536	0.024
Body weight (kg) & White blood cells (×10^3/μL)	-0.539	-2.395	0.031
Body weight (kg) & CD8+CTLA-4+ [%]	-0.539	-2.395	0.031
IgE (mg/dL) & Neutrophils (×10^3/mL)	-0.522	-2.371	0.032
Lymphocytes (×10^3/mL) & CD8+PD-L1+ [%]	-0.506	-2.275	0.038
Hemoglobin (g/dL) & CD8+PD-1+ [%]	0.491	2.185	0.045
Height (cm) & CD19+CTLA-4+ [%]	0.503	2.253	0.040
CD8+PD-1+ [%] & CD19+CTLA-4+ [%]	0.505	2.265	0.039
sPD-L1 (serum) & CD19+PD-L1+ [%]	0.512	2.310	0.036
Hemoglobin (g/dL) & CD19+CTLA-4+ [%]	0.514	2.318	0.035
Monocytes (×10^3/μL) & CD4+PD-1+ [%]	0.518	2.347	0.033
Body weight (kg) & CD8+PD-1+ [%]	0.523	2.295	0.038
Height (cm) & CD8+PD-1+ [%]	0.525	2.388	0.031
IgA (mg/dL) & Monocytes (×10^3/μL)	0.530	2.422	0.029
Monocytes (×10^3/μL) & sPD-L1 (serum)	0.545	2.515	0.024
sPD-1 (serum) & CD8+PD-1+ [%]	0.549	2.544	0.022
Platelets (×10^3/μL) & CD8+CTLA-4+ [%]	0.564	2.643	0.018
CD4+PD-L1+ [%] & CD4+CTLA-4+ [%]	0.583	2.782	0.014
Body weight (kg) & CD19+CTLA-4+ [%]	0.586	2.707	0.017
IgM (mg/dL) & CD4+PD-1+ [%]	0.596	2.877	0.012
Body weight (kg) & Hemoglobin (g/dL)	0.607	2.855	0.013
IgM (mg/dL) & IgE (mg/dL)	0.620	3.061	0.008
Neutrophils (×10^3/mL) & Monocytes (×10^3/μL)	0.657	3.374	0.004
sPD-1 (serum) & sCTLA-4 (serum)	0.694	3.729	0.002
Red blood cells (mL/μL) & Hemoglobin (g/dL)	0.703	3.825	0.002
Age (years) & Hemoglobin (g/dL)	0.736	4.213	0.001
White blood cells (×10^3/μL) & Monocytes (×10^3/μL)	0.770	4.677	<0.001
Height (cm) & Hemoglobin (g/dL)	0.792	5.020	<0.001
Age (years) & Body weight (kg)	0.850	6.050	<0.001
Age (years) & Height (cm)	0.892	7.640	<0.001
White blood cells (×10^3/μL) & Neutrophils (×10^3/mL)	0.902	8.090	<0.001
Height (cm) & Body weight (kg)	0.919	8.749	<0.001

Table S3. Significant correlations (Pearson's r) in girls with CL/P (study group)

Variable pair	r	t (N–2)	p-value
CD4+CTLA-4+ [%] & CD19+CTLA-4+ [%]	-0.621	-2.860	0.013
Birth weight (g) & CD4+CTLA-4+ [%]	-0.611	-2.558	0.027
IgA (mg/dL) & CD4+PD-1+ [%]	-0.579	-2.558	0.024
Height (cm) & Lymphocytes (×10³/mL)	-0.564	-2.460	0.029
Hemoglobin (g/dL) & Monocytes (×10³/µL)	-0.559	-2.433	0.030
Age (years) & Lymphocytes (×10³/mL)	-0.556	-2.415	0.031
Body weight (kg) & Lymphocytes (×10³/mL)	-0.550	-2.282	0.042
Monocytes (×10³/µL) & CD4+PD-1+ [%]	-0.539	-2.307	0.038
Height (cm) & Red blood cells (mL/µL)	-0.531	-2.262	0.042
IgG (mg/dL) & Neutrophils (×10³/mL)	0.518	2.183	0.048
IgG (mg/dL) & White blood cells (×10³/µL)	0.525	2.224	0.044
Neutrophils (×10³/mL) & CD8+PD-1+ [%]	0.539	2.309	0.038
IgG (mg/dL) & sCTLA-4 (serum)	0.546	2.352	0.035
sPD-L1 (serum) & sCTLA-4 (serum)	0.557	2.419	0.031
Platelets (×10³/µL) & sPD-1 (serum)	0.561	2.442	0.030
White blood cells (×10³/µL) & CD8+PD-1+ [%]	0.600	2.704	0.018
Red blood cells (mL/µL) & CD8+PD-1+ [%]	0.603	2.724	0.017
CD4+PD-1+ [%] & CD8+PD-L1+ [%]	0.611	2.781	0.016
IgA (mg/dL) & IgG (mg/dL)	0.643	3.026	0.010
CD4+PD-L1+ [%] & CD4+CTLA-4+ [%]	0.657	3.143	0.008
Red blood cells (mL/µL) & Hemoglobin (g/dL)	0.660	3.171	0.007
Birth weight (g) & IgA (mg/dL)	0.718	3.421	0.006
sPD-1 (serum) & CD8+CTLA-4+ [%]	0.775	4.422	0.001
White blood cells (×10³/µL) & Neutrophils (×10³/mL)	0.864	6.195	<0.001
Age (years) & Body weight (kg)	0.878	6.357	<0.001
Height (cm) & Body weight (kg)	0.930	8.731	<0.001
Age (years) & Height (cm)	0.965	13.228	<0.001

Table S4. Significant correlations in children with CL/P born by vaginal delivery (N group)

Variable pair	r	t (N–2)	p-value
Hemoglobin (g/dL) & CD19+ B cells (%)	-0.694	-3.200	0.008
IgM (mg/dL) & Red blood cells (mL/µL)	-0.646	-2.810	0.017
Red blood cells (mL/µL) & CD19+ B cells (%)	-0.644	-2.790	0.018
CD8+ T cells (%) & sPD-L1 (serum)	-0.632	-2.704	0.021
Birth weight (g) & IgG (mg/dL)	-0.606	-2.285	0.048
CD19+ B cells (%) & CD8+PD-L1+ (%)	-0.588	-2.410	0.035
sPD-1 (serum) & sCTLA-4 (serum)	0.555	2.212	0.049
Monocytes (×10³/µL) & CD19+ B cells (%)	0.570	2.302	0.042
sPD-L1 (serum) & sCTLA-4 (serum)	0.571	2.309	0.041
IgG (mg/dL) & CD19+PD-L1+ (%)	0.593	2.445	0.033
IgA (mg/dL) & Monocytes (×10³/µL)	0.607	2.416	0.036
Age (years) & CD4+ T cells (%)	0.608	2.537	0.028
Platelets (×10³/µL) & CD8+ T cells (%)	0.615	2.589	0.025
IgA (mg/dL) & CD3+ T cells (%)	0.650	2.707	0.022
CD45+ leukocytes (%) & CD4+ T cells (%)	0.687	3.134	0.010

CD4+PD-1+ (%) & CD8+PD-L1+ (%)	0.698	3.231	0.008
IgA (mg/dL) & CD4+ T cells (%)	0.699	3.094	0.011
CD8+ T cells (%) & CD8+PD-1+ (%)	0.720	3.439	0.006
CD3+ T cells (%) & CD8+ T cells (%)	0.725	3.494	0.005
IgA (mg/dL) & White blood cells ($\times10^{3}$/μL)	0.727	3.351	0.007
CD3+ T cells (%) & CD4+ T cells (%)	0.742	3.668	0.004
CD45+ leukocytes (%) & CD3+ T cells (%)	0.747	3.729	0.003
IgA (mg/dL) & Neutrophils ($\times10^{3}$/mL)	0.769	3.807	0.003
Age (years) & Body weight (kg)	0.785	4.005	0.002
Red blood cells (mL/μL) & Hemoglobin (g/dL)	0.798	4.389	0.001
IgA (mg/dL) & CD45+ leukocytes (%)	0.825	4.619	0.001
Age (years) & Height (cm)	0.865	5.726	<0.001
Neutrophils ($\times10^{3}$/mL) & Monocytes ($\times10^{3}$/μL)	0.882	6.193	<0.001
White blood cells ($\times10^{3}$/μL) & Monocytes ($\times10^{3}$/μL)	0.895	6.667	<0.001
Height (cm) & Body weight (kg)	0.954	10.108	<0.001
White blood cells ($\times10^{3}$/μL) & Neutrophils ($\times10^{3}$/mL)	0.962	11.611	<0.001

Table S5. Significant correlations in children with CL/P born by caesarean section (CC group)

**Variable pair**	**r**	**t (N–2)**	**p-value**
Hemoglobin (g/dL) & Monocytes ($\times10^{3}$/μL)	-0.773	-5.028	<0.001
Height (cm) & White blood cells ($\times10^{3}$/μL)	-0.688	-3.913	0.001
Body weight (kg) & White blood cells ($\times10^{3}$/μL)	-0.652	-3.441	0.003
Body weight (kg) & Lymphocytes ($\times10^{3}$/mL)	-0.593	-2.946	0.009
Height (cm) & Neutrophils ($\times10^{3}$/mL)	-0.574	-2.891	0.010
Red blood cells (mL/μL) & Monocytes ($\times10^{3}$/μL)	-0.540	-2.644	0.017
CD4+ T cells (%) & CD19+PD-L1+ (%)	-0.500	-2.380	0.029
IgG (mg/dL) & CD4+CTLA-4+ (%)	-0.498	-2.371	0.030
Age (years) & White blood cells ($\times10^{3}$/μL)	-0.496	-2.354	0.031
CD19+ B cells (%) & CD8+PD-L1+ (%)	-0.495	-2.347	0.031
Body weight (kg) & Neutrophils ($\times10^{3}$/mL)	-0.489	-2.242	0.040
Height (cm) & Lymphocytes ($\times10^{3}$/mL)	-0.467	-2.178	0.044
White blood cells ($\times10^{3}$/μL) & sPD-L1 (serum)	0.456	2.113	0.050
Platelets ($\times10^{3}$/μL) & CD8+CTLA-4+ (%)	0.460	2.134	0.048
Hemoglobin (g/dL) & CD3+ T cells (%)	0.460	2.139	0.047
Monocytes ($\times10^{3}$/μL) & CD19+PD-L1+ (%)	0.462	2.150	0.046
IgA (mg/dL) & sPD-L1 (serum)	0.463	2.155	0.046
CD45+ leukocytes (%) & CD4+PD-1+ (%)	0.470	2.192	0.043
Birth weight (g) & IgG (mg/dL)	0.470	2.194	0.042
sPD-L1 (serum) & CD4+PD-1+ (%)	0.472	2.208	0.041
Hemoglobin (g/dL) & CD19+CTLA-4+ (%)	0.478	2.244	0.038
Red blood cells (mL/μL) & CD8+PD-1+ (%)	0.482	2.268	0.037
sPD-1 (serum) & CD8+PD-L1+ (%)	0.482	2.271	0.036
CD45+ leukocytes (%) & CD3+ T cells (%)	0.489	2.314	0.033
Platelets ($\times10^{3}$/μL) & sCTLA-4 (serum)	0.491	2.325	0.033
Height (cm) & Hemoglobin (g/dL)	0.495	2.351	0.031
IgM (mg/dL) & CD19+CTLA-4+ (%)	0.503	2.399	0.028
Platelets ($\times10^{3}$/μL) & CD4+ T cells (%)	0.505	2.414	0.027

Height (cm) & IgE (mg/dL)	0.518	2.496	0.023
Red blood cells (mL/μL) & CD19+CTLA-4+ (%)	0.518	2.497	0.023
CD45+ leukocytes (%) & CD8+ T cells (%)	0.523	2.529	0.022
White blood cells (×10³/μL) & Monocytes (×10³/μL)	0.542	2.662	0.016
Hemoglobin (g/dL) & CD8+PD-1+ (%)	0.546	2.685	0.016
IgM (mg/dL) & CD8+PD-1+ (%)	0.569	2.850	0.011
IgE (mg/dL) & CD8+PD-1+ (%)	0.572	2.875	0.011
sPD-L1 (serum) & CD19+PD-L1+ (%)	0.589	3.009	0.008
White blood cells (×10³/μL) & Lymphocytes (×10³/mL)	0.610	3.173	0.006
CD45+ leukocytes (%) & sPD-L1 (serum)	0.611	3.178	0.005
CD4+PD-L1+ (%) & CD4+CTLA-4+ (%)	0.632	3.359	0.004
Age (years) & Hemoglobin (g/dL)	0.658	3.604	0.002
Birth weight (g) & IgA (mg/dL)	0.700	4.045	0.001
sPD-1 (serum) & sCTLA-4 (serum)	0.711	4.163	0.001
Red blood cells (mL/μL) & Hemoglobin (g/dL)	0.744	4.586	<0.001
CD3+ T cells (%) & CD8+ T cells (%)	0.781	5.151	<0.001
Age (years) & Body weight (kg)	0.821	5.743	<0.001
Age (years) & Height (cm)	0.825	6.024	<0.001
White blood cells (×10³/μL) & Neutrophils (×10³/mL)	0.882	7.735	<0.001
Height (cm) & Body weight (kg)	0.937	10.758	<0.001

Supplementary Methods S1. Blood collection, sample processing, storage conditions, and assay organization.

Blood collection and pre-analytical conditions

Peripheral venous blood was collected in the morning, in a fasting state, during scheduled outpatient visits. Children with clinically active viral, bacterial, or fungal infections at the time of eligibility assessment were excluded from the study. Furthermore, participants who had received antibiotic therapy within the three months prior to blood collection were not eligible for the study. Biological material was collected into EDTA-containing tubes—intended for peripheral blood count analysis and immune cell immunophenotyping—and into tubes for serum separation to measure soluble checkpoint molecules.

Sample Processing for Flow Cytometry

Peripheral blood samples collected in EDTA tubes were used for complete blood count analysis and multiparameter immunophenotyping by flow cytometry. Fresh blood samples were analyzed within 24 hours of collection and maintained under controlled laboratory conditions until processing.

Immunophenotyping was performed using fluorochrome-conjugated monoclonal antibodies directed against lineage-specific markers, including anti-CD45 Alexa Fluor 700 (clone 2D1, cat. no. 368514), anti-CD3 PerCP (clone SK7, cat. no. 344814), anti-CD4 BV421 (clone RPA-T4, cat. no. 300532), anti-CD8 BV605 (clone SK1, cat. no. 344742), and anti-CD19 FITC (clone HIB19, cat. no. 302206). Immune checkpoint expression was assessed using anti-PD-1 (CD279) APC (clone EH12.2H7, cat. no. 329908), anti-PD-L1 (CD274) PE (clone 29E.2A3, cat. no. 329706), and anti-CTLA-4 (CD152) PE (clone L3D10, cat. no. 349906). All monoclonal antibodies were obtained from BioLegend (San Diego, CA, USA).

Following antibody incubation, erythrocytes were lysed using a dedicated lysing buffer (BD Biosciences, Franklin Lakes, NJ, USA), and the remaining cells were washed before cytometric acquisition.

Immunophenotyping was performed using a CytoFLEX LX flow cytometer (Beckman Coulter, Brea, CA, USA). Before sample acquisition, daily instrument quality control was performed using CytoFLEX Ready-to-Use Daily QC Fluorospheres (Beckman Coulter, cat. no. C65719). Fluorescence-minus-one (FMO) controls were included for immune checkpoint markers to ensure consistent discrimination between marker-positive and marker-negative cell populations.

A uniform gating strategy was applied to all samples. Following the exclusion of cellular debris and doublets, lymphocytes were identified based on forward- and side-scatter characteristics. T lymphocytes were identified as CD3+ cells and subsequently subdivided into CD3+CD4+ and CD3+CD8+ T-lymphocyte populations, whereas B lymphocytes were identified as CD19+ cells. Within each defined lymphocyte population, the proportions of cells expressing PD-1, PD-L1, and CTLA-4 were determined and expressed as percentages of checkpoint-positive cells within the corresponding parent population.

Flow cytometric data were analyzed using Kaluza Analysis software version 2.1 (Beckman Coulter). The same gating strategy and criteria for determining marker positivity were applied consistently throughout the study.

Serum preparation and storage

Blood intended for the measurement of soluble checkpoint molecules was collected into tubes suitable for serum separation. After collection, the samples were allowed to clot for approximately 30 minutes at room temperature. Serum separation was performed within 2–4 hours of blood collection. Subsequently, the samples were centrifuged at 1500 × g for 10 minutes at room temperature. The resulting serum was transferred to appropriately labeled tubes designed for biological sample storage. Samples were stored at −80°C until the assays for soluble immune checkpoint molecule concentrations were performed.

ELISA assay setup

The following human-specific ELISA kits were used: Human PD-1 (CD279) ELISA Kit (Invitrogen, cat. no. BMS2214; analytical sensitivity: 1.14 pg/mL; assay range: 2.34–150 pg/mL), Human PD-L1 (CD274) ELISA Kit (Invitrogen, cat. no. BMS2327; analytical sensitivity: <0.6 pg/mL; assay range: 4.69–300 pg/mL), and Human CTLA-4 (CD152) ELISA Kit (Invitrogen, cat. no. BMS276; analytical sensitivity: 0.13 ng/mL; assay range: 0.16–10 ng/mL). All kits were obtained from Invitrogen, Thermo Fisher Scientific (Waltham, MA, USA). All assays followed a standardized laboratory procedure, including reagent preparation, calibration curve generation, sample incubation, washing steps, and detection of the enzymatic reaction. Absorbance was measured at a wavelength of 450 nm using a Victor 3 microplate reader (PerkinElmer), with correction at the reference wavelength if required by the specific kit procedure.

The concentrations of the analyzed molecules were calculated using the appropriate calibration curves, accounting for sample dilution factors where applicable.
